# Definition of ‘close contacts’ in leprosy studies: protocol for a scoping review

**DOI:** 10.12688/f1000research.123862.1

**Published:** 2022-07-20

**Authors:** Maya Ronse, Claudia Nieto-Sanchez, Sien De Coninck, Kristien Verdonck, Koen Peeters Grietens

**Affiliations:** 1 Department of Public Health, Institute of Tropical Medicine, Antwerp, Antwerp, 2000, Belgium

**Keywords:** Leprosy, close contacts, scoping review.

## Abstract

Despite difficulties to document transmission pathways (assumed to be airborne), increased risk of leprosy infection has been shown for individuals living in close contact with patients. However, variations in the concept of ‘close contacts’ are used in different settings and studies. We conduct this review to identify criteria of space (location, geographical variables, distance, indoor vs outdoor), time (including frequency and duration), physical exposure (skin to skin, sexual), and relationship (familial, occupational, social) involved in the definition of ‘close contacts’ in leprosy studies. We expect this review to provide an overview of the (lack of) conceptualization of this term and its variations across settings. Primary studies and reviews are eligible for inclusion in this review. The main source of records will be the PubMed interface. Secondary searches will be conducted in Google Scholar, as well as through the reference lists of selected publications. The search strategy is based on the combination of the condition of interest (leprosy) and the concept under study (‘contact’). The findings of this review will be presented using thematic narrative synthesis, tables, and figures. The protocol is written in line with the Prisma Extension for Scoping reviews (PRISMA-ScR).

## Introduction

Despite difficulties to document transmission pathways (assumed to be airborne), increased risk of leprosy infection has been shown for individuals living in *close contact* with patients. The World Health Organisation (WHO) defines *close contact* as “a person having close proximity to a leprosy patient for a prolonged duration. Such persons are considered ‘exposed’ to leprosy and may or may not have been infected. ‘Prolonged duration’ is typically defined as having been in contact with an untreated patient for 20 hours per week for at least three months in a year, e.g. family members, neighbours, friends, school children in same class; co-workers in same office, etc”. ^1^ However, variations of this definition are used in different settings and studies.

Clustering of leprosy cases within households (often referred to as ‘household contacts’) has been documented, ^2^^–^^4^ as well as occurrence of new cases at close geographical distance from previous leprosy cases. ^5^ People living in the same household or at close distance are frequently linked through either social activities or networks, ^6^ raising the question whether the “distance” someone lives from an index case determines the risk of infection or whether it acts as a proxy for other explanatory variables that are more directly associated with leprosy risk, such as types and conditions of close human contacts. ^7^^,^^8^ Similar to other infectious diseases, ^9^ duration of contact has also been considered a criterion to determine risk of exposure.

We conduct this review to identify criteria of space (location, geographical variables, distance, indoor vs outdoor), time (including frequency and duration), physical exposure (skin to skin, sexual), and relationship (familial, occupational, social) involved in the definition of ‘close contacts’ in the context of risk of leprosy. We expect this review to provide an overview of the conceptualization of this term and its variations across settings.

This review is part of the study “Improving leprosy prevention strategies by integrating social network analysis with spatial and molecular epidemiology data of Mycobacterium leprae in the Comoros”, supported by ITM’s Structural Research Funding, and funded by the Flemish Ministry of Economy, Sciences and Innovation (EWI).

### Objectives

The central goal of this review is to identify definitions of ‘close contacts’ used in the description of risk for leprosy transmission, as well as specific criteria of space, time, physical exposure, and relationship employed in these definitions.

## Methods

### Eligibility criteria

Records will be included in the review if they meet all the following criteria:

• Reports of primary studies or review articles (not opinion papers); and
• Using the word ‘contact’ in relation to risk of leprosy, and
• Including a definition of ‘contact’ in relation to risk of leprosy infection

Inter and extra-domiciliary exposures will be included. We expect definitions of contacts to include different criteria to establish risk in relation to space, time, physical contact, and relationships.

The review will consider persons of any age and sex, residing in leprosy-endemic areas. Definitions might include participants recruited in the community (active case finding) or in health establishments (passive case finding); they may be symptomatic or asymptomatic.

### Information sources

PubMed interface will be used for the primary search, without any a priori restrictions to language or date. A limited search of Google Scholar as a secondary source will also be conducted. We intend to screen the reference lists of included records (especially review papers) and contact experts in the field to check if we have missed any potentially relevant records.

### Search strategy

The search strategy is based on the combination of two concepts: the condition of interest and the concept of ‘contact’. The Boolean operators “AND” and “OR” will be used to combine search terms. Table 1 summarizes the planned search syntax for PubMed. The same general strategy will be used to search in Google Scholar.

**Table 1.** Planned search syntax for PubMed.

“leprosies”[All Fields] OR “leprosy”[MeSH Terms] OR “leprosy”[All Fields] OR (“mycobacterium leprae”[MeSH Terms] OR (“mycobacterium”[All Fields] AND “leprae”[All Fields]) OR “mycobacterium leprae”[All Fields]) OR (“mycobacterium leprae”[MeSH Terms] OR (“mycobacterium”[All Fields] AND “leprae”[All Fields]) OR “mycobacterium leprae”[All Fields] OR “m leprae”[All Fields]) OR (“mycobacterium lepromatosis”[Supplementary Concept] OR “mycobacterium lepromatosis”[All Fields] OR “mycobacterium lepromatosis”[All Fields]) OR (“m”[All Fields] AND “lepromatosis”[All Fields])
AND
(contact)

### Data extraction (selection and coding)

All retrieved records will be imported into COVIDENCE. Duplicate records will be identified and excluded using COVIDENCE and Mendeley. Two reviewers will independently select full-text papers to be included in this review. Discordances will be solved through discussion with the review team. Two reviewers will extract data items into a data extraction form in COVIDENCE that will include the categories included in Table 2. This table will be pilot tested on five papers, and then refined based on the results of the pilot. Data extraction will only consider published records; no contact with authors is planned.

**Table 2.** Categories of data items to be considered in the preliminary data extraction form.

Category	Data items
Record	Journal and year of publication, first author and affiliation
Setting	Study aim, location, type of setting (urban, rural)
Conceptual frame	Definition of ‘contact’
	Methods used for the identification of index cases and contacts
	Parameters of space (location, distance, geographical, intra/extra domiciliary, in/out-door)
	Parameters of time (frequency, duration)
	Parameters of relationship (genetic, familial, occupational, other social types)
	Parameters of physical contact (skin to skin, sexual)
	Other conditions
Intervention (if existing)	Contact tracing, screening, prevention, treatment, prophylaxis, active case detection, passive case detection

### Strategy for data synthesis

Thematic narrative synthesis will be our main method of data reporting. Results will be inserted in each one of the categories specified in the final data extraction form. Information extracted from each manuscript will be indicated in summary tables. If considered useful, additional figures will be created.

### Registration

This protocol is registered in F1000Research. The protocol has been developed in line with the Prisma Extension for Scoping reviews (PRISMA-ScR) recommendations. ^10^

### Planning

*Timeframe*

• Protocol publication: July 2022
• Search, selection, data extraction and synthesis: July 2022 – October 2022
• Writing of review paper: November 2022 – January 2023

*Study status*

In preparation of this protocol, preliminary searches have been conducted (mostly to grasp the extent of the available literature). However, formal reviewing activities had not started yet.

## Data availability

No data are associated with this article.

## References

[1] World Health Organization (WHO): *Leprosy/Hansen disease: contact tracing and post-exposure prophylaxis.* World Health Organization;2020. Regional Office for South-East Asia.

[2] Weng X Xing Y Liu J : Molecular, ethno-spatial epidemiology of leprosy in China: Novel insights for tracing leprosy in endemic and non endemic provinces. *Infect. Genet. Evol.* 2013;14:361–368. 10.1016/j.meegid.2012.12.009 23291419 PMC3668695

[3] Blok DJ Vlas SJ de Fischer EAJ : Chapter Two - Mathematical Modelling of Leprosy and Its Control. Anderson RM Basáñez MGBT-A in P , editors. *Mathematical Models for Neglected Tropical Diseases: Essential Tools for Control and Elimination, Part A.* Academic Press;2015; pp.33–51. 10.1016/bs.apar.2014.12.002

[4] Ortuno-Gutierrez N Baco A Braet S : Clustering of leprosy beyond the household level in a highly endemic setting on the Comoros, an observational study. *BMC Infect. Dis.* 2019;19:501. 10.1186/s12879-019-4116-y 31174481 PMC6556052

[5] Kendall C Kerr LRFS Miranda JGV : A social network approach for the study of leprosy transmission beyond the household. *Trans. R. Soc. Trop. Med. Hyg.* 2022;116:100–107. 10.1093/trstmh/trab071 34015825

[6] Ashamalla L : Impact of Leprosy on Family and Intimate Relationships. *Int. J. Dermatol.* 1987;26:305–307. 10.1111/j.1365-4362.1987.tb00194.x 3610436

[7] Feenstra SG Nahar Q Pahan D : A qualitative exploration of social contact patterns relevant to airborne infectious diseases in northwest Bangladesh. *J. Health Popul. Nutr.* 2013;31:424–434. 10.3329/jhpn.v31i4.19976 24592583 PMC3905636

[8] Feenstra SG Nahar Q Pahan D : Social contact patterns and leprosy disease: a case-control study in Bangladesh. *Epidemiol. Infect.* 2013;141:573–581. 10.1017/S0950268812000969 22583511 PMC9167658

[9] Mossong J Hens N Jit M : Social Contacts and Mixing Patterns Relevant to the Spread of Infectious Diseases. *PLoS Med.* 2008;5:e74. 10.1371/journal.pmed.0050074 18366252 PMC2270306

[10] Tricco AC Lillie E Zarin W : PRISMA Extension for Scoping Reviews (PRISMA-ScR): Checklist and Explanation. *Ann. Intern. Med.* 2018;169:467–473. 10.7326/M18-0850 30178033

