# Supplementary material for: Definition of ‘close contacts’ in leprosy studies: protocol for a scoping review
Source: F1000Res. 2022 Jul 20;11:808. [Version 1] doi: 10.12688/f1000research.123862.1 (PMC10345595; doi:10.12688/f1000research.123862.1)
Supplement: Peer review history

# Peer review: Reviewer response for version 1

**DOI:** 10.5256/f1000research.136014.r172160

**Published:** 2023-07-13

**Authors:** Veronica Schmitz

**Affiliations:** 1 Oswaldo Cruz Foundation, Rio de Janeiro, Brazil

The manuscript entitled "Definition of ‘close contacts’ in leprosy studies: protocol for a scoping review" by Ronse et al. describes the literature review that the authors plan to carry out with a highly relevant topic which is the definition of contacts in leprosy. The close contact for leprosy is defined by WHO, however many different authors provide different definitions and found different results of what is a close contact or household contact."

 The article has straightforward question and a search strategy defined.

 Minor points:

 Methods:

1. The eligibility criteria: I would suggest to define study inclusion and exclusion criteria separately.
2. Why don't you also use EMBASE, Web of Science, Scopus, LILACS, Virtual Health Library or Cochrane Library databases?
3. It is not clear if the development process will include the characterization of each selected study, evaluation of their quality, identification of important concepts, and comparison of statistical analyses used.
4. Is the planning/Timeframe updated?

Is the study design appropriate for the research question?

Yes

Is the rationale for, and objectives of, the study clearly described?

Yes

Are sufficient details of the methods provided to allow replication by others?

Yes

Are the datasets clearly presented in a useable and accessible format?

Not applicable

Reviewer Expertise:

Immunology of leprosy, clinical research

I confirm that I have read this submission and believe that I have an appropriate level of expertise to confirm that it is of an acceptable scientific standard.

# Peer review: Reviewer response for version 1

**DOI:** 10.5256/f1000research.136014.r172157

**Published:** 2023-06-13

**Authors:** Vimal Kumar

**Affiliations:** 1 National JALMA Institute for Leprosy and Other Mycobacterial Diseases, Agra, Uttar Pradesh, India

Summary:

 The authors conducted this review to identify criteria of space (location, geographical variables, distance, indoor vs outdoor), time (including frequency and duration), physical exposure (skin to skin, sexual), and relationship (familial, occupational, social) involved in the definition of ‘close contacts’ in the context of risk of leprosy. They expect that this review will provide an overview of the conceptualization of this term and its variations across settings.

 The proposal has good scientific and technical merit as the definition of contacts must be accepted in similar sense worldwide as still some ambiguity persists with the term 'contacts'.

 Comments:

 a. Is the rationale for, and objectives of, the study clearly described?

 Comment: Yes, the rationale for, and objectives of, the study clearly described.

 As the objective is to conceptualize the term on the different criteria of space, time, physical exposure, and relationship the correlation of the above facts will help in reaching the final conclusion in a better way.

 b. Is the study design appropriate for the research question?

 Comment: Yes the study design is appropriate for the research question. The idea is innovative and has good novelty sense.

 c. Are sufficient details of the methods provided to allow replication by others?

 Comment: Yes, sufficient details of the methods have been provided to allow replication by others. As the assay will evaluated on the different criterion, it will be very beneficial for the leprosy experts to come to a common conclusion for the leprosy contacts.

 d. Are the datasets clearly presented in a useable and accessible format?

 Comment: The datasets have been presented in a useable and accessible format but I have one suggestion regarding improvement of the same.

 Suggestion: Instead of two, three reviewers should independently select full-text papers to be included in this review for the significant results.

Is the study design appropriate for the research question?

Yes

Is the rationale for, and objectives of, the study clearly described?

Yes

Are sufficient details of the methods provided to allow replication by others?

Yes

Are the datasets clearly presented in a useable and accessible format?

Partly

Reviewer Expertise:

Leprosy, tuberculosis, animal experimentation

I confirm that I have read this submission and believe that I have an appropriate level of expertise to confirm that it is of an acceptable scientific standard.

# Peer review: Reviewer response for version 1

**DOI:** 10.5256/f1000research.136014.r155569

**Published:** 2022-12-05

**Authors:** Millawage Supun Dilara Wijesinghe

**Affiliations:** 1 Health Promotion Bureau, Colombo, Sri Lanka

Thank you for the opportunity to review this paper, focusing on a crucial topic. This protocol for a scoping review is well-written and provides adequate details about the proposed methods. Furthermore, the methods seem well justified.

 I have a few minor suggestions which will improve the paper further.

**Abstract **

- The abstract should be structured as mentioned in the PRISMA-ScR guidelines ^1^.
- It is always better to have MeSH terms as keywords.

**Introduction**

- In the introduction section, it is better to have what are the problems associated with not having a proper definition of 'close contact'.
- It is also best to describe current discrepancies in the definition of contact in leprosy.
- The authors can further justify the rationale for undertaking this review in the present context.

**Methods**

- Search strategy
  - What about grey literature? Are you planning to include them?
- Table 2 - Setting - location - at what level (country level or below)?
- Intervention (If existing) - is this for identification of the index case or identification of contacts?

**Protocol and Registration**

- Please include the protocol's web address or registration number.

Is the study design appropriate for the research question?

Yes

Is the rationale for, and objectives of, the study clearly described?

Yes

Are sufficient details of the methods provided to allow replication by others?

Yes

Are the datasets clearly presented in a useable and accessible format?

Not applicable

Reviewer Expertise:

Leprosy, Public Health, Health Promotion, Occupational Health

I confirm that I have read this submission and believe that I have an appropriate level of expertise to confirm that it is of an acceptable scientific standard, however I have significant reservations, as outlined above.

## References

[1] : PRISMA Extension for Scoping Reviews (PRISMA-ScR): Checklist and Explanation. *Ann Intern Med* .2018;169(7) : 10.7326/M18-0850 467-473 10.7326/M18-0850 30178033
